# Putrescine Intensifies Glu/GABA Exchange Mechanism and Promotes Early Termination of Seizures

**DOI:** 10.3390/ijms23158191

**Published:** 2022-07-25

**Authors:** Zsolt Kovács, Serguei N. Skatchkov, Zsolt Szabó, Saif Qahtan, Miguel P. Méndez-González, Christian J. Malpica-Nieves, Misty J. Eaton, Julianna Kardos, László Héja

**Affiliations:** 1 Department of Biology, Savaria University Centre, ELTE Eötvös Loránd University, Károlyi Gáspár tér 4, 9700 Szombathely, Hungary; kovacs.zsolt@sek.elte.hu; 2 Department of Physiology, Universidad Central del Caribe, Bayamon, PR 00960, USA; serguei.skatchkov@uccaribe.edu (S.N.S.); 415cmalpica@uccaribe.edu (C.J.M.-N.); 3 Department of Biochemistry, Universidad Central del Caribe, Bayamon, PR 00960, USA; miguel.mendez3@upr.edu (M.P.M.-G.); misty.eaton@uccaribe.edu (M.J.E.); 4 Functional Pharmacology Research Group, Institute of Organic Chemistry, Research Centre for Natural Sciences, Magyar Tudósok Körútja 2, 1117 Budapest, Hungary; szabo.zsolt@ttk.hu (Z.S.); saif.qahtan@qu.edu.iq (S.Q.); kardos.julianna@ttk.hu (J.K.); 5 Hevesy György PhD School of Chemistry, ELTE Eötvös Loránd University, Pázmány Péter sétány 1/A, 1117 Budapest, Hungary; 6 College of Science, University of Al-Qadisiyah, Al-Diwaniyah 58001, Iraq; 7 Natural Sciences Department, University of Puerto Rico in Aguadilla, Aguadilla, PR 00604, USA; 8 Department of Science and Technology, Antilles Adventist University, Mayagüez, PR 00681, USA

**Keywords:** WAG/Rij rat model of absence epilepsy, low-[Mg^2+^] hippocampal slice model of temporal lobe epilepsy, polyamine putrescine, ictal discharge duration, astroglial Glu/GABA exchange mechanism, astrocytes in diseases

## Abstract

Endogenous anticonvulsant mechanisms represent a reliable and currently underdeveloped strategy against recurrent seizures and may recall novel original therapeutics. Here, we investigated whether the intensification of the astroglial Glu-GABA exchange mechanism by application of the GABA precursor putrescine (PUT) may be effective against convulsive and non-convulsive seizures. We explored the potential of PUT to inhibit spontaneous spike-and-wave discharges (SWDs) in WAG/Rij rats, a genetic model of absence epilepsy. Significant shortening of SWDs in response to intraperitoneally applied PUT has been observed, which could be antagonized by blocking GAT-2/3-mediated astrocytic GABA release with the specific inhibitor SNAP-5114. Direct application of exogenous GABA also reduced SWD duration, suggesting that PUT-triggered astroglial GABA release through GAT-2/3 may be a critical step in limiting seizure duration. PUT application also dose-dependently shortened seizure-like events (SLEs) in the low-[Mg^2+^] in vitro model of temporal lobe epilepsy. SNAP-5114 reversed the antiepileptic effect of PUT in the in vitro model as well, further confirming that PUT reduces seizure duration by triggering glial GABA release. In accordance, we observed that PUT specifically reduces the frequency of excitatory synaptic potentials, suggesting that it specifically acts at excitatory synapses. We also identified that PUT specifically eliminated the tonic depolarization-induced desynchronization of SLEs. Since PUT is an important source of glial GABA and we previously showed significant GABA release, it is suggested that the astroglial Glu-GABA exchange mechanism plays a key role in limiting ictal discharges, potentially opening up novel pathways to control seizure propagation and generalization.

## 1. Introduction

Epileptic phenomena are mostly featured by recurrent seizure dynamics highlighting initiation and termination of ictal discharges. These dynamics present spreading synchrony of EEG discharges before their termination [1,2,3,4,5,6]. Synchronized network activity, however, may be either the cause or the symptom of the genesis, progress, generalization, and termination of the bursting system’s behavior [7,8,9,10,11,12].

We have previously shown that increased network activity during seizures emerges a negative feedback process, the Glu-GABA exchange mechanism, by which the increased intracellular [Na^+^] due to astrocytic Glu uptake reverses the glial GABA transporters that are known to operate close to their reversal potential [13,14]. The released GABA activates extrasynaptic GABA receptors and generates tonic inhibition proportional to the increased network activity [14]. Importantly, we also showed that a specific blockade of this anticonvulsant mechanism increases the duration of seizure-like events (SLEs) in the in vitro low-[Mg^2+^] model of temporal lobe epilepsy [14], suggesting that the Glu-GABA exchange mechanism contributes to the termination of seizures. Since the released astrocytic GABA is synthesized from the polyamine putrescine (PUT) [14], it is reasonable to regard the PUT-GABA conversion as a potential intervention point through which the inhibitory feedback can be modified. Indeed, we found that blocking the major catabolic conversion of PUT to spermidine (SPD), therefore increasing the PUT pool available for GABA synthesis, drastically eliminated seizures (spike-and-wave discharges: SWDs) in the in vivo absence epilepsy model WAG/Rij rats [15]. It has been demonstrated previously that SWDs are evoked by imbalanced excitatory and inhibitory mechanisms, leading to hyperexcitability in the somatosensory cortex (cortical focus of absence epilepsy genesis: cortical focus theory) [16]. Consequently, and according to this theory, intensified GABA release from astrocytes may be able to attenuate excessive cortical hyperexcitability in the somatosensory cortex resulting decrease in absence epileptic seizures.

We are confident that the realm of the astroglial Glu-GABA exchange mechanism enhanced the possibility of understanding the role of disinhibition in seizure development and recognizing novel targets along with the potential of furthering antiepileptics [13,14,15,17,18,19,20,21,22,23]. Therefore, in this study, we investigated whether the intensification of the astroglial Glu-GABA exchange mechanism by exogenous application of the GABA precursor PUT [24] may be relevant in seizure dynamics.

## 2. Results

### 2.1. Exogenously Applied PUT Reduces Seizure Duration in the In Vivo WAG/Rij Rat Model of Absence Epilepsy

We have previously shown that PUT is a significant source of GABA synthesis in astrocytes during epileptiform activity and that astrocytic GABA is released in response to Glu uptake, causing tonic inhibition [13,14]. We have also demonstrated that specific blockade of the astrocytic GABA transporters GAT-2/3 by SNAP-5114 increases the duration of epileptiform activity in the in vitro low-[Mg^2+^] model of temporal lobe epilepsy [14]. In addition, we observed that inhibition of PUT conversion to SPD and the subsequently increased PUT to GABA conversion diminishes seizures in the in vivo WAG/Rij rat model of absence epilepsy [15]. Conclusively, it is conceivable that PUT may serve as an anticonvulsant.

**Figure 1:**
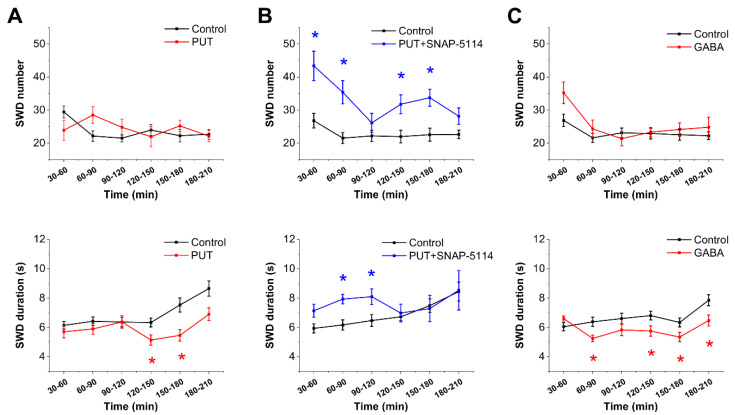
Activation of the Glu/GABA exchange mechanism by exogenous PUT reduces the duration of spike-wave discharges in the in vivo WAG/Rij rat model of absence epilepsy. (**A**–**C**) Effect of PUT (25 mg/kg, N = 10) (**A**), PUT (25 mg/kg) + SNAP-5114 (20 mg/kg, N = 8) (**B**) and GABA (300 mg/kg, N = 10) (**C**) on SWD number (top) and average SWD duration (bottom). Asterisks denote significant difference (*p* < 0.05) from control. PUT reduces SWD duration after approximately 2 h, which is completely antagonized by the specific blocker of astrocytic GAT-2/3 GABA transporters, SNAP-5114. GABA also reduces SWD duration, but without delay, indicating that PUT may need to be converted before exerting its anticonvulsant action.

To investigate whether exogenously applied PUT may offer antiepileptic activity, we first applied PUT (25 mg/kg, i.p.) in WAG/Rij rats, a genetic model of human absence epilepsy. In this model, PUT did not affect the frequency of seizures (number of SWDs) but significantly shortened the duration of individual SWDs between 120 and 180 min following application (Figure 1A), qualifying PUT as being antiepileptic. It is to note that a significant delay, approximately 120 min, was observed between PUT application and the emergence of its antiepileptic effect (Figure 1A), suggesting that an enzymatic conversion, such as the PUT to GABA catabolism, was needed to bring up the antiepileptic action.

Importantly, the reduction in SWD duration by PUT could be antagonized by blocking GAT-2/3-mediated astrocytic GABA release by applying the specific irreversible inhibitor SNAP-5114 (20 mg/kg, i.p.) (Figure 1B). In addition, SNAP blockade also increased the frequency of seizures and the SWD duration in the first 120 min when PUT did not yet exert its effect (Figure 1B), suggesting that GAT-2/3-mediated GABA release was already in place even without PUT application.

The above effects proposed that the antiepileptic effect of PUT is due to the PUT to GABA conversion and subsequent GAT-2/3-mediated GABA release. This hypothesis implies that activation of extrasynaptic GABA receptors induces an antiepileptic effect. Since it is controversial whether GABA*ergic* activation is anti- or pro-epileptic in absence epilepsy [8,15,18,25,26], we directly applied saturating concentration of GABA (300 mg/kg, i.p.) to WAG/Rij rats. Indeed, GABA significantly reduced SWD duration (Figure 1C), similar to PUT. However, in contrast to the delayed reduction in SWD duration by PUT, GABA shortened SWDs almost immediately, suggesting that an enzymatic conversion was not needed in this case, in line with the implied PUT to GABA conversion following PUT application.

In conclusion, the observed antiepileptic effect of applied PUT can be assigned to PUT uptake, catabolism to GABA, and subsequent release of GABA through astroglial GAT-2/3. It is expected that the awareness of the role the astroglial Glu/GABA exchange mechanisms played in seizure duration opens up novel pathways to epilepsy research and shall contribute to improved therapeutics.

### 2.2. PUT Dose-Dependently Reduces Seizure Duration in the In Vitro Low-[Mg^2+^] Model of Temporal Lobe Epilepsy

**Figure 2:**
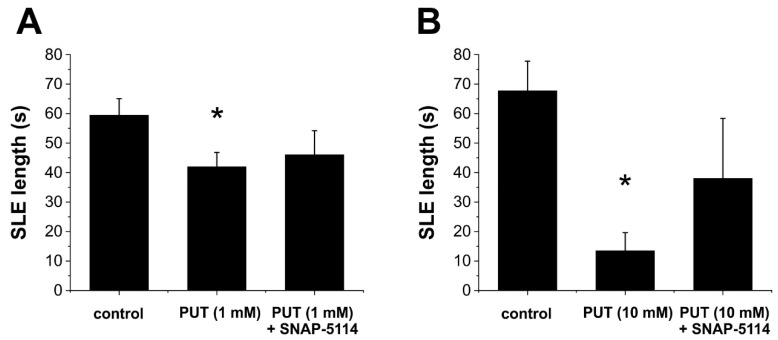
Putrescine dose-dependently reduces the length of seizure-like events in the in vitro low-[Mg^2+^] model of temporal lobe epilepsy. (**A**) Effect of exogenously applied PUT (1 mM, N = 9) and PUT (1 mM) + SNAP-5114 (100 µM, N = 5) on the duration of seizure-like events (SLEs). Asterisk denotes significant difference from control (*p* = 0.02). PUT + SNAP-5114 application does not differ significantly from control (*p* = 0.17). (**B**) Effect of exogenously applied PUT (10 mM, N = 10) and PUT (10 mM) + SNAP-5114 (100 µM, N = 5) on the duration of seizure-like events (SLEs). Asterisk denotes significant difference from control (*p* = 0.0004). PUT + SNAP-5114 application does not differ significantly from control (*p* = 0.25).

Next, we validated the in vivo observed antiepileptic activity of exogenous PUT in the low-[Mg^2+^] in vitro model of temporal lobe epilepsy. We observed that, similar to the results obtained in the absence epilepsy model, PUT (1 mM, N = 9) also reduced the duration of SLEs in the in vitro temporal lobe epilepsy model (*p* = 0.02, Figure 2A). Importantly, the antiepileptic effect of PUT could be reversed by applying SNAP-5114 (100 µM), a specific blocker of the glial GABA transporter subtypes GAT-2/3 (Figure 2A).

In addition, we investigated the dose-response of the PUT effect on SLEs. Increasing the PUT concentration to 10 mM (N = 10) further reduced average SLE duration compared to control (*p* = 0.0004, Figure 2B). Moreover, in 6 of 10 slices, 10 mM PUT completely eliminated SLE appearance. This antiepileptic PUT effect was not observed when 100 µM SNAP-5114 was present. SLEs were generated in all slices and average SLE duration did not decrease compared to control (*p* = 0.25, N = 5, Figure 2B). Instead, SLE duration in the presence of PUT (10 mM) + SNAP-5114 (100 µM) was significantly increased compared to PUT (10 mM) application alone (*p* = 0.001, Figure 2B), demonstrating that PUT reduces seizure duration by increasing glial GABA release through the Glu-GABA exchange mechanism.

### 2.3. Glial GABA Release Following PUT Application Specifically Reduces Excitatory Signals

**Figure 3:**
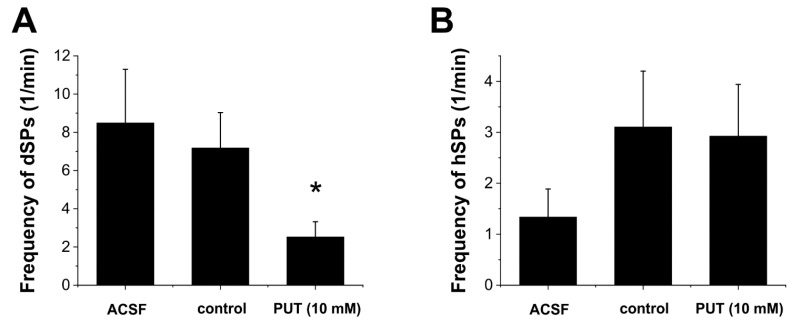
Putrescine specifically reduces depolarizing synaptic potentials in the in vitro low-[Mg^2+^] model of temporal lobe epilepsy. Effect of normal ACSF (ACSF), low-[Mg^2+^] ACSF (control), and low-[Mg^2+^] ACSF with addition of 10 mM putrescine (PUT 10 mM) on the frequency of depolarizing (**A**) and hyperpolarizing (**B**) spontaneous synaptic potentials in pyramidal cells of CA1 hippocampus (N = 8). Asterisk denotes significant difference from control (*p* = 0.036).

To further confirm the mechanism by which PUT application reduces epileptiform activity, we measured whether inhibitory or excitatory synaptic activity is affected by PUT. We recorded miniature spontaneous synaptic potentials in CA1 pyramidal cells in the low-[Mg^2+^] in vitro epilepsy model (Figure 3). We observed that 10 mM PUT produced a significant (*p* = 0.036, N = 8) depression of spontaneous depolarizing potentials (dSPs) (Figure 3A). In contrast, inhibitory synaptic potentials (hSPs) were not affected (*p* = 0.9, N = 8, Figure 3B). The specific decrease in dSPs may be related to the astrocytic release of PUT-derived GABA in the in vitro low-[Mg^2+^] model of temporal lobe epilepsy and also correlates with decreasing of SLE length (Figure 2B) produced by 10 mM of PUT.

### 2.4. PUT Specifically Limits Tonic Phase of SLEs in the In Vitro Low-[Mg^2+^] Model of Temporal Lobe Epilepsy

**Figure 4:**
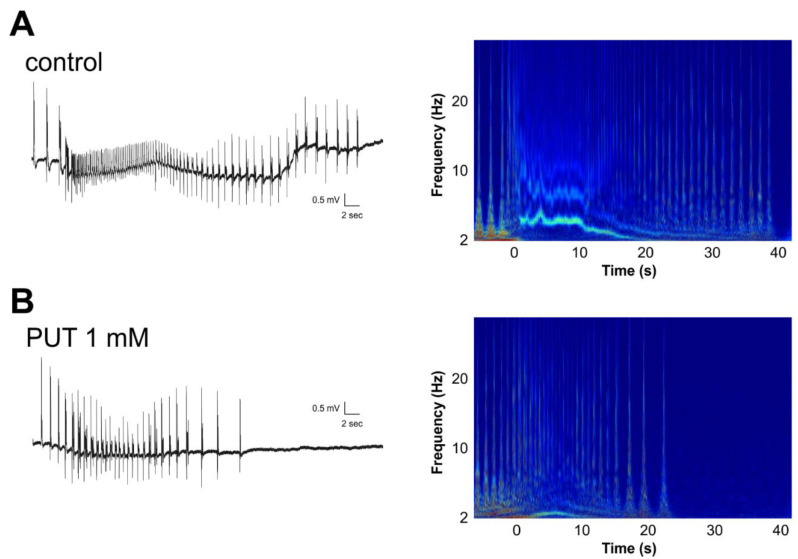
Putrescine impacts the high-frequency tonic phase of seizure-like events in the in vitro low-[Mg^2+^] model of temporal lobe epilepsy. Wavelet analysis (right) shows that compared to the control condition in low-[Mg^2+^] ACSF (**A**), the reduction in SLE duration by 1 mM PUT (**B**) corresponds to the disappearance of the high-frequency tonic phase. Zero time point marks SLE onset on the wavelet representation.

Next, we analyzed the dynamics of SLEs under control conditions and in the presence of 1 mM PUT. Wavelet analysis revealed that the decreasing characteristic high frequencies specifically observed in the tonic phase of SLEs (Figure 4A) were completely missing in the presence of PUT (Figure 4B), suggesting that PUT specifically affects depolarization-induced tonic desynchronization [27]. In addition, the relationship of high-frequency spiking patterns with/without PUT indicates unchanged development of synchronization. In short, PUT makes SLEs shorter by promoting desynchronization.

## 3. Discussion

**Figure 5:**
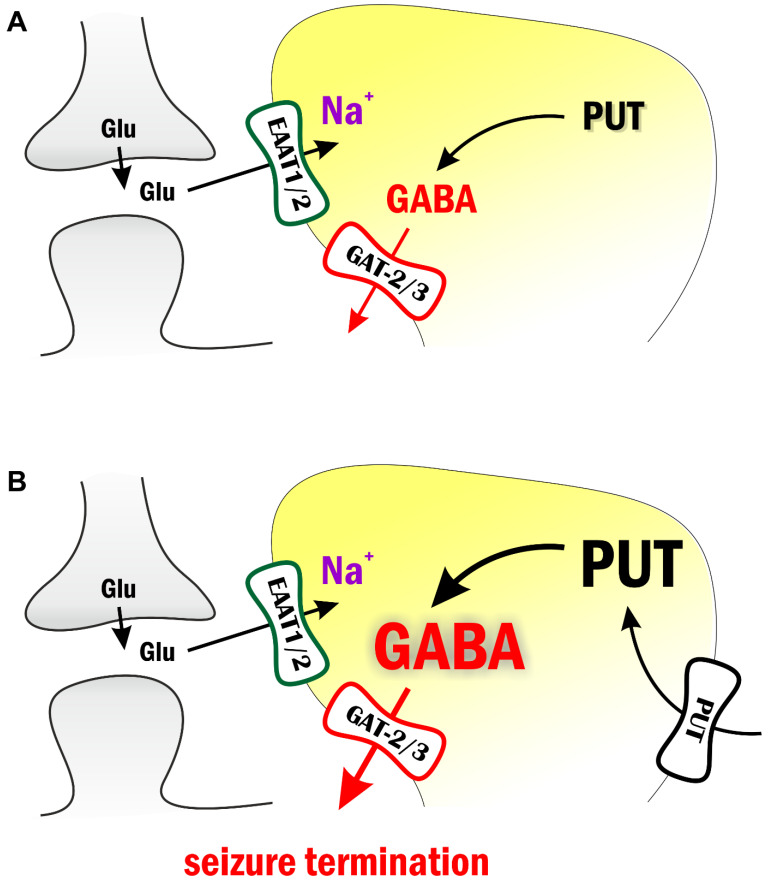
Exogenous putrescine application increases astrocytic GABA production and contributes to seizure termination. (**A**) During intense network activity periods, glutamate transport to astrocytes leads to increased intracellular [Na^+^], which in turn reverses astrocytic GABA transporters GAT-2/3. The released GABA activates extrasynaptic GABA receptors and increases tonic inhibition. Importantly, astrocytic GABA is synthesized from putrescine (PUT). (**B**) Exogenously applied PUT is transported into astrocytes and leads to increased astrocytic GABA concentration. The resulting further increased tonic inhibition terminates seizures.

We discuss the antiepileptic effects of GABA precursor PUT applied in the WAG/Rij rat model of absence epilepsy and in the low-[Mg^2+^] in vitro model of temporal lobe epilepsy. We showed that the application of exogenous PUT reduced epileptic activity in both models (Figure 5). Importantly, similar PUT effects were observed in models of both convulsive (temporal lobe epilepsy) and non-convulsive (absence epilepsy) epilepsies, confirming the universal presence and importance of the astroglial Glu-GABA exchange mechanism [13,14] that functions as an endogenous antiepileptic mechanism. Our findings present the idea that the endogenous astroglial Glu-GABA exchange mechanism can be intensified with added PUT, also implying its cellular uptake and subsequent catabolism to GABA (Figure 5). Significantly, the anticonvulsant PUT appears to switch ictal discharges over interictal spikes. The observed phenomena may control spatio-temporal dynamics of seizures similar to the “inhibitory restraint” function of interneurons [28,29,30,31]; however, using astrocytes instead of interneurons to provide the GABA*ergic* inhibition. Within this context, the excitatory core of ictal discharges remains local by inhibitory restraints. It is this inhibitory halo that prevents propagation of the excitatory core and seizure generalization in the end [31]. The ability of astrocytes to produce significant and highly localized inhibitory constrain is confirmed by (i) the observation that SNAP-5114, a specific blocker of astrocytic GAT-2/3, could reverse the antiepileptic effect of PUT (Figure 2) and (ii) only excitatory synaptic potentials were reduced by PUT application (Figure 3) By turning excitation into inhibition, the astroglial Glu-GABA exchange mechanism not only drives against ictal fatigue comprising impaired metabolism, mitochondrial malfunctioning and cellular energy depletion [5,15,32,33] but can prevent propagation and generalization of seizures. We conclude that the enhancement of GABA release through astroglial GAT-2/3 transporters entitles PUT with therapeutic potential in absence epilepsy [34,35,36].

## 4. Materials and Methods

### 4.1. Animals

Animals were kept and used in accordance with standard ethical guidelines and approved by the local Animal Care Committee, the Government Office for Pest County (reference numbers PEI/001/3671-4/2015 and PE/EA/3840-4/2016), the Hungarian Act of Animal Care and Experimentation (1998, XXVIII, section 243), European Communities Council Directive 24 November 1986 (86/609/EEC) and EU Directive 2010/63/EU on the use and treatment of animals in experimental laboratories. The experiments on WAG/Rij rats were approved by the Animal Care and Experimentation Committee of the Eötvös Loránd University (Savaria University Centre) and the National Scientific Ethical Committee on Animal Experimentation (Hungary) under license number VA/ÉBNTF02/85-8/2016 and VA/ÉBÁF-ÁO/00279-4/2021. The experiments on Sprague–Dawley rats were carried out in accordance with the protocol by the UCC Institutional Animal Care and Use Committee (UCC, Bayamon, PR, USA), approved by protocol numbers: #018-2021-05-010 and #018-2021-04-00, approval date: March 2021. All efforts were made to reduce animal suffering and the number of animals used. All animals were housed in groups of 3–4 under standard laboratory conditions (free access to water and food; 12:12 h light-dark cycle, the light was on from 08:00 a.m. to 08:00 p.m.; air-conditioned room at 22 ± 2 °C). In total, 29 Wistar rats and 8 Sprague–Dawley rats were used for the in vitro epilepsy measurements and to determine the effect of PUT on synaptic activity. In addition, 30 WAG/Rij rats were used for the in vivo epilepsy measurements.

### 4.2. Solutions

*Artificial cerebrospinal fluid* (ACSF) contained in mM: 129 NaCl; 3 KCl; 1.6 CaCl_2_; 1.8 MgSO_4_; 1.25 NaH_2_PO_4_; 21 NaHCO_3_; 10 glucose. To induce epilepsy, MgSO_4_ was omitted, and 2 mM KCl was added (low-[Mg^2+^] ACSF). In the in vitro experiments, PUT (1 mM) was diluted in low-[Mg^2+^] ACSF. The pH value of 7.4 was not affected by the applied concentration. All solutions were continuously oxygenated (95% O_2_, 5% CO_2_). In the in vivo experiments on WAG/Rij rats, PUT (intraperitoneal/i.p. 25 mg/kg) and GABA (i.p. 300 mg/kg) were dissolved in saline. It was demonstrated previously that 1–30% dimethyl sulfoxide (DMSO) solution did not change the absence epileptic activity in WAG/Rij rats [37]; thus, SNAP-5114 (i.p. 20 mg/kg; TOCRIS, Bristol, UK) was dissolved in 10% DMSO solution. Unless otherwise stated, all drugs were purchased from Sigma-Aldrich, Budapest, Hungary, and Saint Louis, MO, USA.

### 4.3. Slice Preparation

*Rat brain slices for low-[Mg^2+^] epilepsy model measurements.* Hippocampal-entorhinal slices from 10–15-day-old Wistar rats (Toxicoop, Budapest, Hungary) and 25–28-day-old Sprague–Dawley rats of both sexes were prepared. Transverse, 400 μm thick slices from Wistar rats were cut in modified ACSF (75 mM sucrose; 87 mM NaCl; 2.5 mM KCl; 0.5 mM CaCl_2_; 7 mM MgSO_4_; 1.25 mM NaH_2_PO_4_; 25 mM NaHCO_3_; 25 mM glucose) at 4 °C. Slices were incubated in an interface-type chamber in continuously oxygenated ACSF for 1 h at 37 °C followed by incubation in room temperature before performing the experiments. Transverse 250 µm thick brain slices from Sprague–Dawley rats were used to measure spontaneous inhibitory (hSPs) and excitatory (dSPs) synaptic potentials from pyramidal neutrons in stratum pyramidale in the CA1 area of the hippocampus. The slices were dissected in ice-cold ACSF saturated with 95% O_2_ and 5% CO_2_. Slices were cut using a vibratome (VT1000S; Leica, Nussloch, Germany) and incubated for recovery in a standard ACSF solution containing (mM) 127 NaCl, 2.5 KCl, 1 MgCl_2_, 2 CaCl_2_, 1.25 NaH_2_PO_4_, 10 glucose, 26 NaHCO_3_, gassed with 5% CO_2_/95% O_2_, pH 7.4, at 35 °C for 20 min (osmolarity: 305 mOsm/L). After 30 min of total incubation, slices were placed in a recording flow chamber (0.5 mL volume) and superfused continuously with oxygenated ACSF at room temperature (23–24 °C, 1 mL/min). Whole-cell recordings were performed as described previously [15,38,39].

### 4.4. Electrophysiology

*Low-[Mg^2+^] epilepsy model for field potential (FP) recordings*. FP recordings were performed at 31 °C using glass microelectrodes (1 to 4 MΩ), filled with ACSF solution, and inserted in the CA3 *stratum pyramidale*. Signals were recorded with Multiclamp 700A amplifiers (Axon Instruments, Foster City, CA, USA), low-pass filtered at 2 kHz, and digitized at 10 kHz or 20 kHz (Digidata 1320A, Axon Instruments). Recordings were analyzed after high pass filtering at 1 or 2 Hz. Epileptiform activity was induced by switching the perfusing solution (ACSF) to low-[Mg^2+^] ACSF (ACSF with no added MgSO_4_ and KCl elevated to 5 mM).

*Low-[Mg^2+^] epilepsy model for recording spontaneous synaptic excitatory depolarizing (dSP) and inhibitory hyperpolarizing (hSP) potentials*. The patch-clamp electrophysiology recordings were performed in brain slices from pyramidal cells in the CA1 hippocampus. Experiments were performed at 23–24 °C using patch pipettes (5–7 MΩ) filled with an intracellular solution in mM: 105 K-gluconate, 20 K_2_ATP, 10 Phosphocreatine, 0.3 GTP, 4 ATP-MgCl_2_, 10 KCl, 10 HEPES, pH 7.2, osmolarity 301 mOsm. While blowing intracellular solution from the tip, the patch pipettes were inserted in the CA1 stratum pyramidale. When a gigaseal was achieved, the membrane of the pyramidal cell was opened by additional suction, and membrane potential was measured. Keeping current-clamp mode, we further recorded the natural spontaneous electrical activity of the cells while perfusing with different solutions. Signals were recorded with Multiclamp 700B amplifier (Molecular Devices, CA, USA), low-pass filtered at 2 kHz, and digitized at 10 kHz or 20 kHz (Digidata 1440A, Axon Instruments, CA, USA). We used pClamp 10.4 software, and recordings were analyzed after high pass filtering at 1 or 2 Hz. Spontaneous activity was recorded constantly while switching the perfusing solutions. First, normal ACSF solution was applied for 10 min as a control condition. Then low-[Mg^2+^] ACSF for 10 min. The low-[Mg^2+^] ACSF contained 10 µM MgCl_2_ and 2.5 mM KCl. Finally, low-[Mg^2+^] ACSF with 10 mM PUT was applied for a further 10 min.

*EEG recording in the WAG/Rij rat model of absence epilepsy.* Female WAG/Rij rats (8–9 months old, 171–187 g; a breeding colony of WAG/Rij rats at ELTE Savaria University Centre, Szombathely, Hungary) was implanted in an isoflurane-air mixture (2.0–2.5%) anesthesia for in vivo experiments. Screw electrodes were implanted into the bone above the frontal cortex (AP: 2.0 mm and L: 2.1 mm) and parietal cortex (AP: −6.5 mm and L: 2.1 mm) [40] for EEG recording. A ground electrode was placed above the cerebellar cortex, whereas the one-side insulated reference electrode (a 3 × 4 mm stainless steel plate) was implanted under the skin and over the masseter muscle. The plate and electrodes were soldered to a ten-pin socket and fixed to the skull with dentacrylate cement (Ivoclar, Liechtenstein). Lidocaine ointment (5%; EGIS, Hungary) was used for post-operative pain relief. After implantations, all rats were allowed to recover for 2 weeks.

Electroencephalogram (EEG) was recorded by a differential biological amplifier (Bioamp4, Supertech Ltd., Pécs, Hungary), which was attached to a data capture and analysis device (CED 1401 mkII, Cambridge Electronic Design Ltd., Cambridge, UK). The bandwidth of the EEG recording was 0.16 Hz to 150 Hz, and it was sampled at a 1 kHz sampling rate. Handling may evoke stress-induced changes in behavior for about 30 min, which can modify SWD numbers [8,41]. Thus, the evaluation of SWD parameters was carried out between 30 and 210 min of recording period between 03:00 PM and 06:00 PM. Normal grooming and behavior were observed in all animals 20–25 min after the drug administration and the connection of rats to the biological amplifier.

To adapt the WAG/Rij rats to the experimental procedures, all animals were connected to the biological amplifier for 3 days (adaptation period) after 2 weeks recovery period. After the adaptation period, rats were randomly assigned into three groups. To establish averaged control SWD number and SWD time, all rats were i.p. injected twice per day by saline (2 mL/kg body weight/1st injection and, 30 min later, it was followed by the same saline injection/2nd injection) on three-day control period. On the 4th day (after three control days), the first group of animals (N = 10) was injected with i.p. saline (2 mL/kg, 1st injection) and PUT (30 min later; 25 mg/kg in 2 mL/kg saline; 2nd injection). Animals in the second group (n = 8) received SNAP-5114 (i.p. 20 mg/kg in 2 mL/kg 10% DMSO solution, 1st injection) and, 30 min later, PUT (i.p. 25 mg/kg in 2 mL/kg saline; 2nd injection) on the 4th day. In the third group (n = 10), similar treatments to the first group were carried out on the 4th day, but the second i.p. injection contained 300 mg/kg GABA. EEGs were recorded every day.

### 4.5. Data Digitization and Processing

The Clampfit (Axon Instruments) program was used to evaluate electrophysiological data. Recordings were analyzed after high pass filtering at 1 Hz. Being not fully developed, the first SLE in each slice (SLE0) was discarded from data evaluation.

SWDs can be characterized by 7–11 Hz discharge frequency within SWDs, 1–50 s duration, and 0.2–1.0 mV amplitude [41]. Moreover, SWDs contain a train of asymmetric spikes and slow waves starting and ending with sharp spikes, and the average amplitude of SWDs is at least twice as high as the basal EEG activity. Similar to our previous study [15], EEG recordings were split into 30 min sections, and the features of SWDs above were used for automated separation of SWDs in the EEG files (all automatically selected SWDs were confirmed by manual supervision).

All statistical analyses were performed using Matlab, Origin 2021, Sigma Plot, and GraphPad Prism (version 8.4.3 (471), San Diego, CA, USA). Data are reported as mean ± SEM. Significant differences between groups were evaluated using two-way ANOVA. Statistical significance was accepted for *p* < 0.05.

## Data Availability

The data that support the findings of this study are available upon request from the authors.
